# Guidelines for Parkinson’s disease treatment: consensus from the Movement Disorders Scientific Department of the Brazilian Academy of Neurology -motor symptoms

**DOI:** 10.1590/0004-282X-ANP-2021-0219

**Published:** 2022-03-31

**Authors:** Roberta Arb Saba, Débora Palma Maia, Francisco Eduardo Costa Cardoso, Vanderci Borges, Luiz Augusto F. Andrade, Henrique Ballalai Ferraz, Egberto Reis Barbosa, Carlos Roberto de Mello Rieder, Delson José da Silva, Hsin Fen Chien, Tamine Capato, Ana Lúcia Rosso, Carlos Frederico Lima Souza, José Marcelo Ferreia Bezerra, Denise Nicaretta, Orlando Graziani Povoas Barsottini, Clécio Godeiro-Júnior, Lorena Broseghini Barcelos, Rubens Gisbert Cury, Mariana Spitz, Sônia Maria César Azevedo Silva, Marcus Vinicius Della Colletta

**Affiliations:** 1 Universidade Federal de São Paulo, Departamento de Neurologia, Setor de Transtornos do Movimento, São Paulo SP, Brazil. Universidade Federal de São Paulo Departamento de Neurologia Setor de Transtornos do Movimento São Paulo SP Brazil; 2 Hospital do Servidor Público Estadual, Serviço de Neurologia, Setor de Transtornos do Movimento, São Paulo SP, Brazil. Hospital do Servidor Público Estadual Serviço de Neurologia Setor de Transtornos do Movimento São Paulo SP Brazil; 3 Universidade Federal de Minas Gerais, Departamento de Neurologia, Setor de Transtornos do Movimento, São Paulo SP, Brazil. Universidade Federal de Minas Gerais Departamento de Neurologia Setor de Transtornos do Movimento São Paulo SP Brazil; 4 Hospital Israelita Albert Einstein, São Paulo SP, Brazil. Hospital Israelita Albert Einstein São Paulo SP Brazil; 5 Universidade de São Paulo, Faculdade de Medicina, Departamento de Neurologia, Centro de Distúrbios do Movimento, São Paulo SP, Brazil. Universidade de São Paulo Faculdade de Medicina Departamento de Neurologia São Paulo SP Brazil; 6 Universidade Federal de Ciências de Saúde de Porto Alegre, Hospital Santa Casa de Porto Alegre, Porto Alegre RS, Brazil. Universidade Federal de Ciências de Saúde de Porto Alegre Hospital Santa Casa de Porto Alegre Porto Alegre RS Brazil; 7 Hospital das clínicas da Universidade Federal de Goiás, Goiânia GO, Brazil. Hospital das clínicas da Universidade Federal de Goiás Goiânia GO Brazil; 8 Universidade do Estado do Amazonas, Manaus AM, Brazil. Universidade do Estado do Amazonas Manaus AM Brazil; 9 Serviço de Neurologia Prof. Sérgio Novis, Rio de Janeiro RJ, Brazil. Serviço de Neurologia Prof. Sérgio Novis Rio de Janeiro RJ Brazil; 10 Universidade de Pernambuco, Hospital Universitário Oswaldo Cruz, Recife PE, Brazil. Universidade de Pernambuco Hospital Universitário Oswaldo Cruz Recife PE Brazil; 11 Universidade do Estado do Rio de Janeiro, Faculdade de Ciências Médicas, Rio de Janeiro RJ, Brazil. Universidade do Estado do Rio de Janeiro Faculdade de Ciências Médicas Rio de Janeiro RJ Brazil; 12 Universidade Federal do Estado do Rio de Janeiro, Rio de Janeiro RJ, Brazil. Universidade Federal do Estado do Rio de Janeiro Rio de Janeiro RJ Brazil; 13 Universidade Federal do Rio Grande do Norte, Hospital Universitário Onofre Lopes, Natal RN, Brazil. Universidade Federal do Rio Grande do Norte Hospital Universitário Onofre Lopes Natal RN Brazil; 14 Hospital Universitário Pedro Ernesto, Rio de Janeiro RJ, Brazil. Hospital Universitário Pedro Ernesto Rio de Janeiro RJ Brazil

**Keywords:** Parkinson Disease, Antiparkinson Agents, Deep Brain Stimulation, Rehabilitation, Doença de Parkinson, Antiparkinsonianos, Estimulação Encefálica Profunda, Reabilitação

## Abstract

The treatment of Parkinson's disease (PD) is challenging, especially since it is considered highly individualized. The Brazilian Academy of Neurology has recognized the need to disseminate knowledge about the management of PD treatment, adapting the best evidence to the Brazilian reality. Thus, the main published treatment guidelines were reviewed based on the recommendations of group from the Movement Disorders Scientific Department of the Brazilian Academy of Neurology.

## Resumo

O tratamento da doença de Parkinson (DP) constitui um desafio, especialmente por ser considerado muito individualizado. A Academia Brasileira de Neurologia (ABN) identificou a necessidade de disseminar o conhecimento sobre o manejo do tratamento da DP, adaptando as melhores evidências à realidade brasileira. Assim, foi realizada uma revisão sobre as principais orientações de tratamento publicadas, baseada nas recomendações elaboradas por um grupo de especialistas em transtornos do movimento do departamento científico da ABN.

## INTRODUCTION

Parkinson's disease (PD), first described by James Parkinson in 1817, is a neurodegenerative disease characterized by motor (stiffness, bradykinesia, resting tremor and postural instability) and non-motor symptoms (neuropsychiatric, sleep, autonomic, and sensory disorders)^1^.

The control of PD symptoms is done through pharmacological, non-pharmacological, and surgical treatment. The Brazilian Academy of Neurology has recognized the need to disseminate knowledge about PD treatment and adapt the best evidence to the Brazilian reality.

In recent years, a group of specialists from the Scientific Department of Movement Disorders of the Brazilian Academy of Neurology has developed a "Guide of Recommendations for the Treatment of Parkinson's Disease", which had two editions. The constant evolution of therapy and the need to quickly reach the largest number of specialists with updated information led this group to the elaboration of two articles in guideline format. 

The first part of this guideline addresses the management of motor symptoms (MS), and the second part addresses the treatment of non-motor symptoms (NMS).

A literature review was carried out in MEDLINE and Cochrane Library databases from 1989 to 2020.

To elaborate this guideline the following topics were searched in relation to PD:

- Treatment of motor symptoms (early and advanced stages)
- Surgical indications
- Rehabilitation therapies

The classification of studies (four classes) and levels of evidence (four levels) were based on the recommendations of the 2017 Edition of the Clinical Practice Guideline Process Manual of the American Academy of Neurology^2^ (Tables 1 and 2).

**Table 1.** Classification of evidence for therapeutic studies.

Class I	A randomized, controlled clinical trial of the intervention of interest with masked or objective outcome assessment, in a representative population. The **a** to **e** criteria* is also required.
Class II	A randomized controlled clinical trial of the intervention of interest in a representative population with masked or objective outcome assessment that lacks one criterion **a-e** above or a prospective matched cohort study with masked or objective outcome assessment in a representative population that meets **b-e** above.
Class III	All other controlled trials (including well-defined natural history controls or patients serving as own controls) in a representative population, where outcome is independently assessed, or independently derived by objective outcome measurement
Class IV	Studies not meeting Class I, II, or III criteria including consensus or expert opinion.

*a: concealed allocation; b: exclusion/inclusion criteria clearly defined; c: Primary outcome(s) clearly defined; d: adequate accounting for dropouts (with at least 80% of enrolled subjects completing the study) and crossovers with numbers sufficiently low to have minimal potential for bias; e: for noninferiority or equivalence trials claiming to prove efficacy for one or both drugs, the following are also required: The authors explicitly state the clinically meaningful difference to be excluded by defining the threshold for equivalence or noninferiority. The standard treatment used in the study is substantially similar to that used in previous studies establishing efficacy of the standard treatment (e.g., for a drug, the mode of administration, dose and dosage adjustments are similar to those previously shown to be effective). The inclusion and exclusion criteria for patient selection and the outcomes of patients on the standard treatment are comparable to those of previous studies establishing efficacy of the standard treatment.

**Table 2.** Level of recommendation.

A	Established as effective, ineffective, or harmful (or established as useful/predictive or not useful/predictive) for the given condition in the specified population
B	Probably effective, ineffective, or harmful (or probably useful/predictive or not useful/predictive) for the given condition in the specified population
C	Possibly effective, ineffective, or harmful (or possibly useful/predictive or not useful/predictive) for the given condition in the specified population
U	Data inadequate or conflicting; given current knowledge, treatment (test, predictor) is unproven

Note that recommendations can be positive or negative.

The interpretation of the results of the study is based upon a per protocol analysis that takes into account dropouts or crossovers.

## CLASSES OF ANTIPARKINSONIAN DRUGS

Several drugs are used for treatment of PD and classified into dopaminergic and nondopaminergic. The dopaminergic drugs include levodopa, dopaminergic agonists (DA), monoamine oxidase-B enzyme (MAO-B) inhibitors, and catechol-ortho-methyltransferase (COMT) inhibitors. Nondopaminergic drugs are amantadine and anticholinergics.

In Brazil, antiparkinsonian drugs are available on the Public Health System, except for extended release pramipexole, safinamide, and rotigotine.

## DOPAMINERGIC DRUGS

### Levodopa

Levodopa is the primary dopamine precursor and is actively transported from the gut (duodenum and jejunum), and its plasma half-life varies from 50 to 120 minutes. The most significant enzymes involved in levodopa peripheral metabolism are dopa decarboxylase (DDC) and COMT. Levodopa crosses the blood-brain barrier through active transport and is converted to dopamine by DDC in dopaminergic neurons and stored in the synaptic vesicles by vesicular monoamine transporter-2 and released to the synaptic cleft^3^ (Table 3).

**Table 3.** Levodopa formulations available in Brazil.

Levodopa + carbidopa	Tablet, 250mg + 25 mg
Levodopa + benserazide	Tablet, 200 mg + 50 mg
Levodopa + benserazide BD (low dose)	Tablet, 100 mg + 25 mg
Levodopa + benserazide (oral dispersible)	Tablet, 100 mg + 25 mg
Levodopa + benserazide HBS (Hydrodynamically Balanced System)	Capsule, 100 mg + 25 mg
Levodopa + benserazide DR (Dual Release)	Tablet, 200mg + 50 mg

### Dopaminergic agonists (DAs)

DAs act directly on striatal dopamine receptors with preferential affinity for the D2-receptor subfamily and do not depend on dopamine-converting enzymes to work. DAs available in Brazil are bromocriptine, pramipexole, and rotigotine. Pramipexole is available in immediate and extended-release formulation. Rotigotine is formulated in transdermal patches based on silicone^4^.

The main adverse effects of DAs are excessive sleepiness and impulse control disorder. Bromocriptine, which currently has very limited use, presents risks of peritoneal, pleural, and pericardial fibrosis and cardiac valve damage^5^^,^^6^.

### MAO-B inhibitors

MAO-B inhibitors increase extracellular dopamine levels in the striate. The formulations available are: selegiline, rasagiline, and safinamide. Selegiline is metabolized to amphetamine derivatives, while one of metabolite of rasagiline is 1-aminoindan that presents antiparkinsonian action. Rasagiline should not be used in association to fluoxetine and fluvoxamine. Safinamide is a novel reversible MAO-B inhibitor and has both dopaminergic and non-dopaminergic effects (inhibits glutamate release by blocking voltage-dependent sodium and N-type calcium channels). 

### COMT inhibitors

 COMT inhibitors decrease the metabolism of levodopa by increasing its supply to the central nervous system^7^ and then offer more stable levodopa plasma levels^8^. They should not be used as monotherapy but as an add-on drug and must be taken with each single dose of levodopa^9^. In Brazil, the only COMT inhibitor available is entacapone.

## NONDOPAMINERGIC DRUGS

### Amantadine

The probable effect of amantadine is increasing the dopamine release and inhibition on N-methyl-D-aspartate (NMDA) receptors^10^. The main side effects reported are hallucinations, mental confusion, and livedo reticularis^11^.

### Anticholinergics

Anticholinergic drugs act by blocking acetylcholine receptors and aim to reestablish the balance between dopaminergic deficits and striatal cholinergic excess in PD^12^^,^^13^.

The main reason for the decline in the use of anticholinergic in current therapy is closely related to their well-known side effects, especially the increased risk of dementia^14^.

## TREATMENT OF EARLY STAGE PD

Drug treatment of PD must be individualized. There are several therapeutic options. The use of drugs in the early stage of PD was reviewed according to the scientific evidence (Table 4).

**Table 4.** Drugs for early-stage PD -levels of evidence.

	Monotherapy	Adjuvant Therapy
Levodopa	Level A	Level A
Dopaminergic Agonist		
Bromocriptine	Level C -Ineffective	
Pramipexole	Level A	
Rotigotine	Level A	
MAO-B Inhibitors		
Selegiline	Level B	Level B
Rasagiline	Level A	Level B
Safinamide		Level C
Amantadine	Level C	Level C
Anticholinergics	Level B	Level B

## ANTICHOLINERGICS

A 2003 Cochrane review^15^ lists nine heterogeneous studies showing efficacy of anticholinergics compared to placebo, leading to improved motor function, but data specifically regarding some tremor benefits were inconclusive^12^.

For younger patients, anticholinergics can be used and remain “clinically useful”^16^. There are no reports of anticholinergic class I clinical studies for the treatment of early stage of PD. 

Anticholinergics, both as monotherapy and adjuvant therapy, should not be the first choice of treatment because of their high rate of adverse effects.

In conclusion, anticholinergics are probably effective in younger patients and in early stages of PD (Level B).

## AMANTADINE

Despite previous studies showing some effectiveness of amantadine in improving motor function, a 2003 Cochrane review^10^ concluded that there was insufficient evidence to support the efficacy of this drug.

Another pharmacological feature of amantadine is the limited duration of clinical effects. Few nonrandomized studies have shown improvement in motor function, but long duration response has not been not proven^10^.

Only six studies compared amantadine with placebo, either as monotherapy or adjuvant therapy^17^^,^^18^. Double-blind studies had limitations regarding the number of included patients (class III).

### Conclusion

Amantadine is possibly effective in early stage PD (level C).

## MONOAMINOXIDASE-B INHIBITORS

### Selegiline

In the DATATOP study^19^, the use of selegiline reduced the need of using levodopa by about 50% (class I). An extension of this study (class II) showed that the benefit of delaying the use of levodopa was maintained for nine months in the selegiline group, and an improvement in the Unified Parkinson's Disease Rating Scale (UPDRS) scores was observed in these patients compared to the placebo group, although without significance. With the withdrawal of selegiline for two months, the motor scores worsened, indicating a symptomatic effect^19^. 

A meta-analysis of 17 randomized trials^20^ concluded that the early use of selegiline delays the need for levodopa, and when used concomitantly, lower doses are required. A systematic review of the Cochrane Database ^21^ had the same conclusion.

### Rasagiline

The TEMPO study compared the efficacy of rasagiline monotherapy (class I) in two doses (1 and 2 mg) with placebo. There was improvement in the UPDRS and in the quality-of-life scale, showing an effect on PD symptoms^22^. The ADAGIO study (class I) showed a benefit of early-start treatment with rasagiline 1 mg/day versus delayed-start treatment^23^.

A randomized (class I), double-blind, placebo-controlled trial of rasagiline 1 mg/d as an add-on therapy in early PD patients using DA monotherapy (ropinirole or pramipexole) showed a significant improvement in total UPDRS scores in the rasagiline group compared with placebo^24^. A meta-analysis including double-blind placebo-controlled trials confirmed the efficacy of rasagiline as monotherapy or as adjuvant^25^.

### Safinamide

Stocchi et al. (2012) was a 24-week double-blind placebo-controlled trial that included 270 early-stage PD patients receiving a stable dose of a single DA randomized into placebo, 100 mg, and 200 mg of safinamide^26^. The difference between 100 mg/day safinamide and placebo was significant, but the difference between 200 mg safinamide and placebo was not. The reason for the lack of efficacy of the higher dose of safinamide is unknown, but the authors suggested that the higher incidence of discontinuations in the 200 mg safinamide group (21.3% vs. 10% each for safinamide 100 mg and placebo) may have prevented a significant clinical benefit. However, no clinically meaningful differences from placebo were observed for any safety variables and the results were considered exploratory.

Shapira et al. (2013) conducted a 12-month randomized double-blind placebo-controlled trial as pre-planned extension of the Stocchi et al. (2012) study. Of the 227 enrolled patients, only 182 (82%) completed the trial. Patients were randomized to 200 mg safinamide, 100 mg safinamide, or placebo in association with a single DA. The primary endpoint was the period between randomization and an additional drug intervention -an increase in the DA dose, an addition of another DA, levodopa, or another PD treatment, or a drug discontinuation due to the lack of efficacy. The median time to “intervention” was not significantly different between the pooled safinamide groups and placebo (559 and 466 days, respectively; p=0.3342). A post-hoc analysis suggested that 100 mg safinamide could be effective as an add-on treatment for PD, but these results should be considered exploratory only ^27^. 

### Conclusion

Selegiline is probably effective as monotherapy and adjuvant therapy in early-stages PD (level B).

Rasagiline is effective as monotherapy (level A) and associated with DA (level B) in early-stage PD.

Safinamide could be effective as adjuvant therapy in early-stage PD (level C).

## DOPAMINERGIC AGONIST

### Bromocriptine

As for the control of motor symptoms, bromocriptine, when used as monotherapy, does not show evidence of greater benefit in relation to levodopa (Class I)^28^. A study comparing bromocriptine with another DA does not show a greater efficacy (Class I)^29^.

A Cochrane systematic review that analyzed the efficacy and safety of the early combination of bromocriptine and levodopa in delaying the onset of motor complications showed that there is no evidence of the use of this association as a strategy to prevent or delay the onset of motor complications in PD (Class I)^28^.

### Pramipexole

A study carried out in 2000 by the Parkinson’s Disease Study Group compared the use of levodopa with that of pramipexole in the early stages of PD. This was a 2-year prospective, randomized, levodopa-controlled study that used pramipexole as monotherapy. One hundred and fifty patients received levodopa and 150 received pramipexole. The results showed that 53% of the patients who were part of the group using pramipexole required levodopa supplementation, against 39% of the patients using levodopa (Class I)^30^.

Two randomized studies comparing pramipexole with placebo showed improvement in the motor response and in the activities of daily living according to the UPDRS (Class I)^31^^,^^32^. 

The Parkinson Study Group study CALM-PD, published in 2009^33^, evaluated the efficacy and motor complications after six years of pramipexole administration with levodopa in patients in the early stages of PD. This analysis was initially performed with 301 individuals, 151 of whom used pramipexole and 150 used levodopa. After six years, it was observed that the scores of Schwab and England were similar in both groups. Motor complications were more common in the group that used levodopa initially (68.4% vs. 50%). There was no statistically significant difference in the UPDRS scores (Class III).

### Rotigotine

A study published in 2007 compared the safety and efficacy of using the rotigotine patch with placebo in early PD. Participants were randomized to receive either placebo (n=96) or rotigotine (n=181) starting from 2 mg/24 h, titrated weekly to 6 mg/2 4h, and then maintained for 6 months. The results showed a significant decrease in the UPDRS scores, showing that rotigotine when titrated to 6 mg is effective in the treatment of PD in its early stages^34^.

### Conclusion

Bromocriptine is possibly ineffective, as monotherapy, compared with levodopa or another DA in early-stage PD (Level C).

Pramipexole is effective as monotherapy in early-stage PD (level A).

The use of pramipexole in early-stage PD allows the appearance of a lower rate of motor complications (Level A).

Rotigotine is effective as monotherapy in early-stage PD (level A).

## LEVODOPA

The class I study Earlier versus Later Levodopa Therapy in Parkinson Disease (ELLDOPA) using three different doses of levodopa (150, 300, and 600 mg) in early stages. Subjects were randomly assigned to receive placebo or carbidopa-levodopa at a dose of 12.5 and 50 mg three times daily, 25 and 100 mg three times daily, or 50 and 200 mg three times daily, respectively. The doses were increased to the maximum over a period of nine weeks in a blinded fashion. PD patients showed significant improvement of the UPDRS scores after 40 weeks compared with the placebo group^35^. One class I and two class II studies compared levodopa with DA in the early stages of PD. They concluded that levodopa, cabergoline, ropinirole, and pramipexol are effective in the treatment of motor symptoms and improve activity of daily life scores (levodopa was more effective than the DA). The final recommendation of the studies was that both levodopa and DA might be used early in PD.

A class II study of controlled-release levodopa compared to rapid-release levodopa demonstrated that both formulations can be used, and the frequency of motor complications is similar in both types (class II)^36^.

In 2019, the Levodopa in Early Parkinson's Disease (LEAP) study was conducted to investigate whether levodopa had a disease-modifying effect. It was designed as an early-vs. delayed-start study. It included 446 patients observed over 80 weeks, divided into 2 groups: 1) levodopa 300 mg/day for 80 weeks and 2) placebo for 40 weeks followed by levodopa 300 mg/day for another 40 weeks. There was no difference between the groups at the end of the study, demonstrating that early or delayed onset of levodopa does not slow disease progression and that starting treatment at low doses according to patient need is the best clinical practice^37^.

### Conclusion

Levodopa is effective in early-stage PD (level A). 

Levodopa alone is more effective than pramipexole and ropinirole alone in improving motor symptoms (level A).

Controlled-release levodopa is probably not effective to prevent the onset of motor complications (level B).

Higher doses of levodopa are related to higher risk of motor complications, and therefore, so it is recommended to start with the lowest possible doses (level A).

Levodopa is effective as monotherapy or in combination with other antiparkinsonian drugs in early-stage PD.

## TREATMENT OF ADVANCED-STAGE PD

Although motor symptoms in PD are highly responsive to dopaminergic drugs, particularly levodopa, the benefit of the drug during diminished in advanced stages of the disease. At the same time, fluctuations and dyskinesias appear.

### Motor fluctuations

 The most important motor fluctuations observed in advanced-stage PD are the wearing-off phenomenon (WO) (shortening effect), delayed-on (delay of motor effect), and no-on (no motor effect at all)^38^^,^^39^ (Table 5).

**Table 5.** Treatments for motor fluctuations in advanced PD -levels of evidence.

	Fluctuations
Levodopa controlled release	No evidence
Dopaminergic Agonist	
Pramipexole	Level A
Rotigotine	Level A
MAO-B Inhibitors	
Selegiline	Level A
Rasagiline	Level A
Safinamide	Level A
COMT Inhibitors	Level A
Amantadine	Ineffective
STN-DBS	Level A

### Fractioning the total dose of levodopa and dietary orientation

 Due to levodopa’s short half-life, it is recommended to reduce the interval between levodopa doses, preferably without increasing the total daily dose^38^. It is also recommended that patients have an interval of at least one hour between the levodopa intake and a meal so that this kind of regimen overcomes the competition with dietary proteins^39^^,^^40^.

### Controlled-released levodopa 

 There are no controlled studies with enough patients to draw definitive conclusions regarding controlled-released levodopa, since most studies are open-label trials^41^^,^^42^.

 The only controlled-release formulation available in Brazil is levodopa/benserazide. There are few studies with this formulation, and their quality is poor. Levodopa associated with benserazide (immediate and slow release in the same tablet), known as dual release, was tested in 61 patients, and there was a decrease in wearing-off, but the exact time of "off-period" was not quantified. Due to methodological reasons, this study should not be considered conclusive^43^.

### Dopaminergic agonists

Pahwa et al. in 2006 and Stocchi in 2008, through a review of current treatments for fluctuations and dyskinesias, concluded that fluctuations can be minimized by the use of dopaminergic agonists, but dyskinesias cannot^44^^,^^45^.

### Pramipexole

In a 1997 multicenter randomized class I study with 360 patients (181 active, 179 control) followed-up for 32 weeks, 83% of the active group and 78% of the control group completed the study. Off-time decreased by 31% in the active group compared to 7% in the placebo group (p=0.0006). Levodopa dose was reduced in the active group (27%) compared to the placebo group (5%) (p=0.0001)^46^.

Guttman in 1997, in a multicenter, double masked, randomized, parallel group (class II study), 79 patients received pramipexole and 83 received placebo for 40 weeks. The active group had a 15% (2.5 hours) decrease in off-time compared with a 3% reduction in the control group (p=0.007). In the on state, the active group also experienced improvements in the UPDRS (p=0.0006)^47^.

Mizuno et al. in 2003, performed a randomized, three-arm parallel study (placebo, bromocriptine and pramipexole) involving 325 patients with advanced PD who had motor fluctuations and freezing for 12 weeks, and UPDRS scores were significantly lower in the pramipexole (p <0.001) and bromocriptine groups. Apparently, the group using pramipexole had a better response, but the study was unable to define this difference^48^.

A study by Wong et al., 2003, followed 150 patients for 15 weeks in a double-blind, randomized, placebo-controlled, parallel group study (levodopa + placebo versus levodopa + pramipexole) and found that the off period was shorter in the pramipexole group, based on UPDRS on and off scores^49^.

### Rotigotine

Poewe et al. published in 2007 the Clinical Efficacy of Pramipexole and Transdermal Rotigotine in Advanced PD (CLEOPATRA-PD) study of rotigotine in adjunctive treatment with levodopa. In this double-blind, randomized, placebo-controlled study, 395 patients with advanced PD with motor fluctuations were followed for six months. The authors found a reduction in the off period in the treatment group^50^. Lewitt et al., also in 2007, published the Prospective Randomized Evaluation of a New Formulation: Efficacy of Rotigotine study (PREFER study). In this randomized, double-blind, placebo-controlled study, 351 patients with advanced PD and motor fluctuation were divided into three groups (8 mg, 12 mg, and placebo). All patients were taking concomitant levodopa. The authors concluded that rotigotine reduces the off time of PD patients safely and with good tolerability^51^. Lewitt et al. published in 2013 the extension of the two previously cited papers, the CLEOPATRA-PD and PREFER (class I) studies, conducted to evaluate the safety, tolerability, and efficacy of rotigotine after several years of follow-up of patients with advanced PD. In the CLEOPATRA-PD study, 48% of the patients remained under follow-up after four years, while in the PREFER, 45% continued after six years of follow-up. In both studies, the rotigotine dose was up to 16 mg. During the whole follow-up, patients who used rotigotine showed better motor improvement than patients who used placebo, but with a decline in the difference in scores over time. The authors concluded that rotigotine is safe, effective, and well-tolerated after six years of follow-up. However, the data regarding the maintenance of the improvement of the off period were not conclusive^52^. 

### COMT inhibitors

Double-blind studies controlled with COMT inhibitors showed a reduction in the off-period with an increase of one to two hours in the on-period, and most studies with entacapone showed improvement in the UPDRS motor score^53^.

Li et al. published in 2017 a meta-analysis of 14 studies evaluating the use of entacapone in PD motor fluctuations. It was demonstrated that the adjuvant use of entacapone and levodopa was effective in the management of motor fluctuations^54^.

### MAO-B inhibitors

In two major class I trials (LARGO and PRESTO), rasagiline has been shown to reduce off-time by around 1 hour in patients with drug-related motor complications^55^^,^^56^. The objective of the PRESTO and LARGO studies was to determine the efficacy and safety or rasagiline as adjunct therapy for levodopa-treated PD patients with motor fluctuations. These were randomized and placebo-controlled studies, but the LARGO study also compared rasagiline with entacapone. The studies showed that rasagiline was effective and safe in adjunct therapy with levodopa to increase motor fluctuations in advanced PD.

There are two double-blind placebo-controlled studies about safinamide in advanced PD: the SETTLE study (50 to 100 mg/day, 24 weeks) and the Borgohain et al., 2014 study ^57^^,^^58^. The primary measure of effectiveness was the change in the “ON” time without problematic dyskinesia between the beginning and the end of the study. Secondary parameters of effectiveness were the off-time, and the UPDRS II and III and CGI-C scales were used. Both indicated a significant superiority of safinamide at the target doses of 50 and 100 mg/day over placebo, concerning the selected primary and secondary efficacy variables. The on-time effect remained until the end of the 24-month treatment period, with both doses of safinamide better than placebo.

### Conclusion

There is no consensus on the interval between levodopa doses or the time between the meal and levodopa intake.

There is no evidence that the controlled-release levodopa formulations available in Brazil are useful to manage fluctuations in advanced-stage PD patients. In clinical practice, controlled-release levodopa formulations could be indicated to treat or prevent nocturnal and early morning akinesia (level U).

 Dopaminergic agonists are effective in the control of motor fluctuations in advanced-stage PD (level A).

COMT inhibitors are effective to control motor fluctuations in advanced-stage PD (level A).

MAO-B inhibitors are effective to control motor fluctuations in advanced-stage PD (level A).

### Levodopa-induced dyskinesia

 Dyskinesia is characterized by involuntary movements related to levodopa use and may appear during the motor benefit of the levodopa effect (square-wave dyskinesia) or at the peak of the effect (peak-dose dyskinesia). Some patients may present dyskinesia only during the beginning and/or the end of the motor effect of levodopa (diphasic dyskinesia) or during the off period (off dyskinesia)^38^^,^^59^ (Table 6).

**Table 6.** Treatments for dyskinesias in advanced PD -levels of evidence.

	Dyskinesias
Amantadine	Level B
Clozapine	Level U
STN-DBS	Level A

### Levodopa management

There are no high-quality studies examining how levodopa is offered to patients to control dyskinetic movements. In clinical settings, patients with peak-dose or square-wave dyskinesia are advised to take more frequent and lower single doses of levodopa. In diphasic dyskinesia, patients are put on a regimen of enhanced dopaminergic stimulation, either by increasing single levodopa doses or adding dopaminergic drugs (DA, COMT inhibitors or MAO-B inhibitors)^59^.

### Amantadine

In 1998, Verhagen Metman et al. recruited 18 patients for a six-week, double-blind, controlled, crossover study evaluating amantadine at doses ranging from 100 to 400 mg daily and placebo. The authors concluded that amantadine substantially improves dyskinesias induced by levodopa without improving motor symptoms of PD. These benefits were sustained for at least 12 months^60^. Amantadine is capable of ameliorating the dyskinesias caused by levodopa use. Amantadine also significantly decreased the duration of off-periods and improved the quality of life of patients in the on and off periods.

 In 2004, Thomas et al. recruited 40 patients for a 12-month, double-blind, randomized, placebo-controlled study. After 15 to 30 days of treatment with amantadine, there was a significant decrease in dyskinesia scores. According to the study, this effect decreased or disappeared after 3 to 8 months of treatment, but the withdrawal of amantadine led to a significant increase of dyskinesias in 11 patients^61^.

In 2010, Wolf et al. conducted a randomized, double-blinded, placebo-controlled, national multicenter study that included 32 patients already using amantadine for at least one year. The authors claimed that amantadine maintains an anti-dyskinetic effect even many years after its introduction^62^. 

 In 2014, the AMANDYSK study evaluated the effect of withdrawal of amantadine, which was replaced by placebo. The study was carried out on 57 patients with PD and dyskinesia, and the patients were followed-up for three months after the withdrawal of amantadine. It was found that the discontinuation of amantadine significantly worsened dyskinesia compared with patients who were not discontinued^63^.

### Clozapine

 In 2004, in a double-blind placebo-controlled trial of clozapine, Durif et al. showed a significant increase of on-time without dyskinesia in the treatment group compared with placebo^64^. An open naturalistic study evaluated the use of clozapine in dyskinetic patients with or without psychotic symptoms. It was observed an improvement in both symptoms^65^. A limitation related to the chronic use of clozapine is the need for regular hematological exams.

### Conclusion

 There is no consensus about the frequency and doses of levodopa to control induced dyskinesia (level U).

 Amantadine is probably effective for controlling levodopa-induced dyskinesias (level B).

Clozapine is an alternative for patients who do not respond to amantadine or who cannot take amantadine (level U).

## DEEP BRAIN STIMULATION FOR THE TREATMENT OF PD PATIENTS

Current surgical indications for PD include reducing motor fluctuations, off time, dyskinesias, tremor, and improvement of levodopa-responsive symptoms. Deep brain stimulation (DBS) is probably the most critical advance in treatment of PD since the introduction of levodopa. The beneficial effects of DBS on motor symptoms and quality of life (QoL) in advanced PD have been shown in randomized, controlled studies^66^^,^^67^.

An excellent individual outcome after DBS for PD patients will depend on appropriate patient selection, accurate electrode placement in the ideal target area, and effective programming of DBS devices after surgery^68^^,^^69^.

### Patients’ inclusion and exclusion criteria

When deciding whether a patient is a good candidate for surgery, numerous factors must be considered, such as: 

### Symptomatology

The primary DBS indication should be for disabling PD motor complications that are not well-controlled with the best available medical treatment and for refractory tremor^70^^,^^71^.

### Levodopa responsiveness

The levodopa response is reported as the best predictive factor for a positive response to surgery. The levodopa challenge is used to reproduce the patient’s best on-response and determine the responsiveness. Tremor is an exemption because it can respond poorly to levodopa but improves with subthalamic nucleus (STN) DBS surgery^72^. 

Axial symptoms, especially gait disturbances, postural instability, freezing, and speech disturbances that do not respond to the peak dose of Levodopa usually do not respond to surgery. “Off-period” gait freezing can improve with surgery, but “on-period” freezing shows little improvement.

### Disease duration

Patients should have a disease duration of at least five years before being considered for surgery^72^^,^^73^. Findings from the EARLYSTIM trial have shown better results of STN stimulation compared with medical treatment at a mean of 7.5 years after disease onset, when patients are just beginning to experience fluctuations. This study argues for considering DBS earlier than currently used in carefully selected patients when the benefits of the treatment are weighed against the surgical risks^74^ However, for early-stage PD patients without motor complications, there is “insufficient evidence”^75^.

### Age

Although no specific age cutoff has been defined in clinical DBS studies, most studies use age as an exclusion criterion. Most patients presenting the ideal profile for surgery have a relatively young onset of PD and are younger than 70 years old. For older patients, the risk-benefit ratio should consider factors such as comorbidities, cognitive performance, prevalence of levodopa-resistant symptoms, and overall risk of surgical complications^72^^,^^76^.

### Cognitive and psychiatric aspects

A preoperative neuropsychological assessment is mandatory. Regarding cognition, dementia is an absolute contraindication for surgery. There are no clear recommendations for mild cognitive impairment^72^^,^^72^. Surgery is contraindicated in patients with unstable psychiatric conditions until symptoms are adequately managed. Ongoing severe depression with suicidal ideation should also be considered an absolute contraindication to surgery. The relationship between DBS and impulse control disorders (ICD) is controversial. STN-DBS has been identified as an independent risk factor for ICD. However, long-term follow-up of patients who underwent STN-DBS showed that ICD disappeared in most patients and the use of dopamine agonist and dopamine dysregulation syndrome were reduced^77^.

### Preoperative imaging MRI

Severe cortical atrophy increases the risk of postoperative subdural hematomas. Visible structural lesions on imaging findings should be considered absolute contraindications to DBS^72^^,^^77^.

### DBS targets

The two most common DBS targets are the STN and globus pallidus pars interna (GPi). Randomized trials have demonstrated no significant difference in the degree of motor improvement or complications between the two targets (with improvement in motor scores by 25%-60%, measured by UPDRS-III scores)^78^.

STN-DBS can reduce the need for dopamine replacement medications by approximately 50%. Therefore, when the primary goal of surgery is to reduce dopaminergic medications, bilateral STN-DBS procedures should be performed instead of GPi^78^^,^^79^. However, patients with STN-DBS can exhibit decreases in visual-motor processing speed and worsening depression scores compared to patients with GPi-DBS^78^. Therefore, if there is significant concern about cognitive issues, GPi-DBS should be considered, rather than STN (76). Similarly, if there is significant concern about the risk of depression, the GPi target should be selected^78^.

Ventral intermediate nucleus (Vim) DBS improves tremor but has no effect on other symptoms; therefore, Vim DBS should be considered only for severe tremor-dominant PD without other bothersome cardinal parkinsonian symptoms^68^^,^^80^. Other targets such as the pedunculopontine nucleus have been suggested as options for DBS, particularly for gait and balance symptoms; however, no trials meeting evidence-based inclusion criteria have been published to date^75^.

### Conclusion

DBS is an effective therapeutic option for controlling disabling motor fluctuations and dyskinesia (Level A).

Because of the risk for adverse events, the procedure is recommended only after consideration of several pre-operatory factors and an evaluation of the risk-benefit ratio by a specialized multidisciplinary team.

## REHABILITATION IN PD

### Physiotherapy

Physical therapy (PT), which includes gait, posture, transfers, balance, physical capacity, and physical activity, plays a crucial role in the management of axial and motor symptoms of people with PD^75^^,^^81^, ^82^^,^^83^. 

One article showed that in-patient multidisciplinary PT is better than “regular” PT (Class I)^84^. Some class II studies have shown significant improvement in specific parameters such as gait speed and step size using external cues (visual and auditory)^85^^,^^86^, whereas cognitive strategies (internal cues) and sensory cues (external cues) improved gait freezing in PD^87^^,^^88^.

Two studies demonstrated the efficacy of dual task training in PD. The RESCUE^85^ class II randomized clinical trial (RCT) enrolled 153 PD patients who received 3 weeks cued gait training and the authors observed that the use of cues enhanced motor learning in PD. Rochester and colleagues defined motor learning as increased acquisition, automaticity, and retention of cued gait after training^89^. The RESCUE trial also indicated the potential for sustained improvement in gait and dual task performance after training. The other study, the DUALITY trial^90^, compared the efficacy of two dual-task training programs for improving dual-task gait in 121 PD patients. After 6 weeks of at-home physiotherapist-led training, both modalities led to a similar and sustained effect on motor learning (Class I), improving dual task gait velocity without increasing the risk of falls. The importance of dual-task training is also observed in gait freezing. Combining treadmill training with visual and auditory cues had more benefits on gait than cue training alone (Class II)^91^.

Two large trials have demonstrated that balance can be improved with PT interventions^92^^,^^93^. The first study (Class II) included 231 PD patients who were randomized into balance exercises or usual-care control groups. Exercises were deliveried during 40 to 60 minutes, 3 times a week for 6 months. The results demonstrated that PT improved balance. However, risk of falls was not reduced in both groups^92^. The second study (Class II) included 100 mild to moderate-stage PD patients and evaluated the short-term effects of a high-challenge balance training, which incorporates both dual-tasking and PD-specific balance components, compared with usual care. At the end of the program, the between group comparison showed significant improvement on balance and gait performance in the intervention group. The intervention group also improved the performance of the cognitive task while walking compared with the control group. No differences between groups were found for falls^93^.

Recently, a large prospective, single-blind RCT (Class II) investigated the effectiveness of multimodal balance training with and without rhythmical auditory cues in 154 PD patients randomized in 3 groups^94^. Both intervention groups improved balance performance compared to controls (educational program). Multimodal balance training supported by auditory rhythmical cues was more effective and had long-term retention effect (6-months). A secondary subgroup analysis for freezers and non-freezers based on the same study showed that adding rhythmic auditory stimuli to balance training is beneficial for both freezers and non-freezer^95^.

Current physiotherapy guidelines provide no recommendations on the specific approach for advanced stages of PD^82^, since there are few studies targeting this subgroup^96^^,^^97^. Multimodal balance intervention (combined or not with rhythmical auditory cues) may improve balance and gait in patients at advanced stages of PD (H&Y 4) (Class III). 

### Conclusion

Physiotherapy is effective in improving motor and axial symptoms in early and moderate stages of PD (Level A). There are insufficient data to support or refute the effectiveness of physiotherapy in advanced stages of PD (Level U).

### Therapeutic and formalized pattern exercises

The SPARX study (Class I) enrolled 128 de novo patients and compared high-and moderate-intensity treadmill exercises with a wait-list control group. After six month of 3 days per week exercise, the results showed that the high-intensity group, who exercised at 80 to 85% maximum heart rate, had less change in motor symptoms (UPDRS motor score) compared with the usual care group^98^. The Park-in-shape trial (Class I), a home-based study, recruited 130 PD patients in Hoehn & Yahr stage ≤ 2 who were randomized either to exercise on a stationary cycle or stretching at least three times per week. After the 6-month program, the MDS-UPDRS motor score change was smaller in the aerobic group, resulting in a between-group adjusted mean difference of 4.2 points favoring the cycling group^99^.

### Conclusion

Aerobic exercises are effective in attenuating symptoms in PD patients in early and mild stages (Level A). Currently, there are insufficient data to support or refute the effectiveness of aerobic exercise in moderate or advanced stages of PD. (Level U).

### Speech therapy

Studies have suggested a beneficial effect of speech language therapy (SLT) in PD^75^^,^^100^ and a newly published systematic review and meta-analysis study (Class II) assessed the effect of SLT on hypokinetic dysarthria in PD patients. The RCT selected in this review compared different SLT in the treatment of three variables, (sound pressure level, semitone standard deviation, and perceptual intelligibility). Results showed significant differences in favor of SLT for sound pressure level in sustained phonation tasks. Significant results were also observed for sound pressure level and semitone standard deviation in reading tasks. This meta-analysis suggests a beneficial effect of SLT for reducing hypokinetic dysarthria, improving perceptual intelligibility, sound pressure level, and semitone standard deviation in PD^101^.

### Conclusion

Speech therapy is possibly effective in improving voice and dysphagia in PD patients (Level C).

### Occupational therapy

Although occupational therapy (OT) is frequently prescribed in the clinical practice^102^, few articles have been published about this intervention in PD patients. In 2014, Sturkenboom et al. demonstrated the impact of OT in daily activities of PD patients^103^. In this study, 191 patients were randomly assigned to the intervention group (n=124), which consisted of 10 weeks of home-based OT, or to the control group (n=67). The primary outcome was self-perceived performance in daily activities, assessed with the Canadian Occupational Performance Measure (COPM). After 3 months, the intervention group had better scores on the COPM, meaning that patients improved self-perceived performance in daily activities. 

A recent review assessed the efficacy of OT intervention on quality of life in PD (Class II). In total, 15 randomized controlled trials were selected for the systematic review and 4 of these were included in the meta-analysis. Both short follow-up (2 -3 months) and long follow-up (6-12 months) studies showed that OT interventions significantly improved the quality of life of patients with PD. However, the strength of the evidence should be considered moderate because of the limited number of publications available^104^.

Because of the lack of high-quality studies available, further investigations are needed to make firm conclusions about OT efficacy in PD.

### Conclusion

Occupational therapy is probably effective in improving daily life activities in PD patients (Level B).

## References

[B1] Parkinson J (2002). An essay on the shaking palsy. 1817. J Neuropsychiatry Clin Neurosci.

[B2] Gronseth GS, Cox J, Gloss D, Merillat S, Dittman J, Armstrong MJ (2017). 2017 Edition Clinical Practice Guideline Process Manual.

[B3] Contin M, Riva R, Albani F, Baruzzi A (1996). Pharmacokinetic optimisation in the treatment of Parkinson’s disease. Clin Pharmacokinet.

[B4] Hutton JT, Metman LV, Chase TN, Juncos JL, Koller WC, Pahwa R (2001). Transdermal dopaminergic D(2) receptor agonist therapy in Parkinson’s disease with N-0923 TDS: a double-blind, placebo-controlled study. Mov Disord.

[B5] Serratrice J, Disdier P, Habib G, Viallet F, Weiller P-J (2002). Fibrotic valvular heart disease subsequent to bromocriptine treatment. Cardiol Rev.

[B6] Andersohn F, Garbe E (2009). Cardiac and noncardiac fibrotic reactions caused by ergot-and nonergot-derived dopamine agonists. Mov Disord.

[B7] Jankovic J, Stacy M (2007). Medical management of levodopa-associated motor complications in patients with Parkinson’s disease. CNS Drugs.

[B8] Cabreira V, Soares-da-Silva P, Massano J (2019). Contemporary options for the management of motor complications in Parkinson’s disease: updated clinical review. Drugs.

[B9] Rodrigues FB, Ferreira JJ (2017). Opicapone for the treatment of Parkinson’s disease. Expert Opin Pharmacother.

[B10] Crosby N, Deane KH, Clarke CE (2003). Amantadine in Parkinson’s disease. Cochrane Database Syst Rev.

[B11] Connolly BS, Lang AE (2014). Pharmacological treatment of Parkinson disease: a review. JAMA.

[B12] Jabbari B, Scherokman B, Gunderson CH, Rosenberg ML, Miller J (1989). Treatment of movement disorders with trihexyphenidyl. Mov Disord.

[B13] (2002). Anticholinergic therapies in the treatment of Parkinson’s disease. Mov Disord.

[B14] Perry EK, Kilford L, Lees AJ, Burn DJ, Perry RH (2003). Increased Alzheimer pathology in Parkinson’s disease related to antimuscarinic drugs. Ann Neurol.

[B15] Katzenschlager R, Sampaio C, Costa J, Lees A (2003). Anticholinergics for symptomatic management of Parkinson’s disease. Cochrane Database Syst Rev.

[B16] Fox SH (2013). Non-dopaminergic treatments for motor control in Parkinson’s disease. Drugs.

[B17] Walker JE, Albers JW, Tourtellotte WW, Henderson WG, Potvin AR, Smith A (1972). A qualitative and quantitative evaluation of amantadine in the treatment of Parkinson’s disease. J Chronic Dis.

[B18] Walker JE, Potvin A, Tourtellotte W, Albers J, Repa B, Henderson W (1972). Amantadine and levodopa in the treatment of Parkinson’s disease. Clin Pharmacol Ther.

[B19] Parkinson Study Group (1993). Effects of tocopherol and deprenyl on the progression of disability in early Parkinson’s disease. N Engl J Med.

[B20] Ives NJ, Stowe RL, Marro J, Counsell C, Macleod A, Clarke CE (2004). Monoamine oxidase type B inhibitors in early Parkinson’s disease: meta-analysis of 17 randomised trials involving 3525 patients. BMJ.

[B21] Macleod AD, Counsell CE, Ives N, Stowe R (2005). Monoamine oxidase B inhibitors for early Parkinson’s disease. Cochrane Database Syst Rev.

[B22] Parkinson Study Group (2002). A controlled trial of rasagiline in early Parkinson disease: the TEMPO study. Arch Neurol.

[B23] Olanow CW, Rascol O, Hauser R, Feigin PD, Jankovic J, Lang A (2009). A double-blind, delayed-start trial of rasagiline in Parkinson’s disease. N Engl J Med.

[B24] Hauser RA, Silver D, Choudhry A, Eyal E, Isaacson S (2014). ANDANTE study investigators. Randomized, controlled trial of rasagiline as an add-on to dopamine agonists in Parkinson’s disease. Mov Disord.

[B25] Chang Y, Wang L-B, Li D, Lei K, Liu S-Y (2017). Efficacy of rasagiline for the treatment of Parkinson’s disease: an updated meta-analysis. Ann Med.

[B26] Stocchi F, Borgohain R, Onofrj M, Schapira AHV, Bhatt M, Lucini V (2012). A randomized, double-blind, placebo-controlled trial of safinamide as add-on therapy in early Parkinson’s disease patients. Mov Disord.

[B27] Schapira AHV, Stocchi F, Borgohain R, Onofrj M, Bhatt M, Lorenzana P (2013). Long-term efficacy and safety of safinamide as add-on therapy in early Parkinson’s disease. Eur J Neurol.

[B28] Hely MA, Morris JG, Reid WG, O’Sullivan DJ, Williamson PM, Rail D (1994). The Sydney Multicentre Study of Parkinson’s disease: a randomised, prospective five-year study comparing low dose bromocriptine with low dose levodopa-carbidopa. J Neurol Neurosurg Psychiatry.

[B29] Korczyn AD, Brooks DJ, Brunt ER, Poewe WH, Rascol O, Stocchi F (1998). Ropinirole versus bromocriptine in the treatment of early Parkinson’s disease: a 6-month interim report of a 3-year study. 053 Study Group. Mov Disord.

[B30] Parkinson Study Group (2000). Pramipexole vs levodopa as initial treatment for Parkinson disease: a randomized controlled trial. JAMA.

[B31] Safety and efficacy of pramipexole in early Parkinson disease (1997). A randomized dose-ranging study. Parkinson Study Group. JAMA.

[B32] Shannon KM, Bennett JP, Friedman JH (1997). Efficacy of pramipexole, a novel dopamine agonist, as monotherapy in mild to moderate Parkinson’s disease. The Pramipexole Study Group. Neurology.

[B33] Parkinson Study Group CALM Cohort Investigators (2009). Long-term effect of initiating pramipexole vs levodopa in early Parkinson disease. Arch Neurol.

[B34] Watts RL, Jankovic J, Waters C, Rajput A, Boroojerdi B, Rao J (2007). Randomized, blind, controlled trial of transdermal rotigotine in early Parkinson disease. Neurology.

[B35] Fahn S, Oakes D, Shoulson I, Kieburtz K, Rudolph A, Lang A (2004). Levodopa and the progression of Parkinson’s disease. N Engl J Med.

[B36] Koller WC, Hutton JT, Tolosa E, Capilldeo R (1999). Immediate-release and controlled-release carbidopa/levodopa in PD: a 5-year randomized multicenter study. Carbidopa/Levodopa Study Group. Neurology.

[B37] Verschuur CVM, Suwijn SR, Boel JA, Post B, Bloem BR, van Hilten JJ (2019). Randomized delayed-start trial of levodopa in Parkinson’s disease. N Engl J Med.

[B38] Freitas ME, Hess CW, Fox SH (2017). Motor complications of dopaminergic medications in Parkinson’s disease. Semin Neurol.

[B39] Contin M, Martinelli P (2010). Pharmacokinetics of levodopa. J Neurol.

[B40] Barichella M, Marczewska A, De Notaris R, Vairo A, Baldo C, Mauri A (2006). Special low-protein foods ameliorate postprandial off in patients with advanced Parkinson’s disease. Mov Disord.

[B41] Hutton JT, Dippel RL, Bianchine JR, Strahlendorf HK, Meyer PG (1984). Controlled-release carbidopa/levodopa in the treatment of Parkinsonism. Clin Neuropharmacol.

[B42] Pahwa R, Busenbark K, Huber SJ, Michalek D, Hubble JP, Koller WC (1993). Clinical experience with controlled-release carbidopa/levodopa in Parkinson’s disease. Neurology.

[B43] Ghika J, Gachoud JP, Gasser U (1997). Clinical efficacy and tolerability of a new levodopa/benserazide dual-release formulation in parkinsonian patients. L-Dopa Dual-Release Study Group. Clin Neuropharmacol.

[B44] Stocchi F, Tagliati M, Olanow CW (2008). Treatment of levodopa-induced motor complications. Mov Disord.

[B45] Pahwa R, Factor SA, Lyons KE, Ondo WG, Gronseth G, Bronte-Stewart H (2006). Practice Parameter: treatment of Parkinson disease with motor fluctuations and dyskinesia (an evidence-based review): report of the Quality Standards Subcommittee of the American Academy of Neurology. Neurology.

[B46] Lieberman A, Ranhosky A, Korts D (1997). Clinical evaluation of pramipexole in advanced Parkinson’s disease: results of a double-blind, placebo-controlled, parallel-group study. Neurology.

[B47] Guttman M (1997). Double-blind comparison of pramipexole and bromocriptine treatment with placebo in advanced Parkinson’s disease. International Pramipexole-Bromocriptine group. Neurology.

[B48] Mizuno Y, Yanagisawa N, Kuno S, Yamamoto M, Hasegawa K, Origasa H (2003). Randomized, double-blind study of pramipexole with placebo and bromocriptine in advanced Parkinson’s disease. Mov Disord.

[B49] Wong KS, Lu C-S, Shan D-E, Yang C-C, Tsoi TH, Mok V (2003). Efficacy, safety, and tolerability of pramipexole in untreated and levodopa-treated patients with Parkinson’s disease. J Neurol Sci.

[B50] Poewe WH, Rascol O, Quinn N, Tolosa E, Oertel WH, Martignoni E (2007). Efficacy of pramipexole and transdermal rotigotine in advanced Parkinson’s disease: a double-blind, double-dummy, randomised controlled trial. Lancet Neurol.

[B51] LeWitt PA, Lyons KE, Pahwa R (2007). SP 650 Study Group. Advanced Parkinson disease treated with rotigotine transdermal system: PREFER Study. Neurology.

[B52] LeWitt PA, Boroojerdi B, Surmann E, Poewe W (2013). SP716 Study Group; SP715 Study Group. Rotigotine transdermal system for long-term treatment of patients with advanced Parkinson’s disease: results of two open-label extension studies, CLEOPATRA-PD and PREFER. J Neural Transm.

[B53] Widnell KL, Comella C (2005). Role of COMT inhibitors and dopamine agonists in the treatment of motor fluctuations. Mov Disord.

[B54] Li J, Lou Z, Liu X, Sun Y, Chen J (2017). Efficacy and safety of adjuvant treatment with entacapone in advanced Parkinson’s disease with motor fluctuation: a systematic meta-analysis. Eur Neurol.

[B55] Parkinson Study Group (2005). A randomized placebo-controlled trial of rasagiline in levodopa-treated patients with Parkinson disease and motor fluctuations: the PRESTO study. Arch Neurol.

[B56] Rascol O, Brooks DJ, Melamed E, Oertel W, Poewe W, Stocchi F (2005). Rasagiline as an adjunct to levodopa in patients with Parkinson’s disease and motor fluctuations (LARGO, Lasting effect in Adjunct therapy with Rasagiline Given Once daily, study): a randomised, double-blind, parallel-group trial. Lancet.

[B57] Cattaneo C, Sardina M, Bonizzoni E (2016). Safinamide as add-on therapy to levodopa in mid-to late-stage Parkinson’s disease fluctuating patients: post hoc analyses of studies 016 and SETTLE. J Parkinsons Dis.

[B58] Borgohain R, Szasz J, Stanzione P, Meshram C, Bhatt M, Chirilineau D (2014). Randomized trial of safinamide add-on to levodopa in Parkinson’s disease with motor fluctuations. Mov Disord.

[B59] Espay AJ, Morgante F, Merola A, Fasano A, Marsili L, Fox SH (2018). Levodopa-induced dyskinesia in Parkinson disease: current and evolving concepts. Ann Neurol.

[B60] Metman LV, Del Dotto P, van den Munckhof P, Fang J, Mouradian MM, Chase TN (1998). Amantadine as treatment for dyskinesias and motor fluctuations in Parkinson’s disease. Neurology.

[B61] Thomas A, Iacono D, Luciano AL, Armellino K, Di Iorio A, Onofrj M (2004). Duration of amantadine benefit on dyskinesia of severe Parkinson’s disease. J Neurol Neurosurg Psychiatry.

[B62] Wolf E, Seppi K, Katzenschlager R, Hochschorner G, Ransmayr G, Schwingenschuh P (2010). Long-term antidyskinetic efficacy of amantadine in Parkinson’s disease. Mov Disord.

[B63] Ory-Magne F, Corvol J-C, Azulay J-P, Bonnet A-M, Brefel-Courbon C, Damier P (2014). Withdrawing amantadine in dyskinetic patients with Parkinson disease: the AMANDYSK trial. Neurology.

[B64] Durif F, Debilly B, Galitzky M, Morand D, Viallet F, Borg M (2004). Clozapine improves dyskinesias in Parkinson disease: a double-blind, placebo-controlled study. Neurology.

[B65] Gomide L, Kummer A, Cardoso F, Teixeira AL (2008). Use of clozapine in Brazilian patients with Parkinson’s disease. Arq Neuropsiquiatr.

[B66] Deuschl G, Schade-Brittinger C, Krack P, Volkmann J, Schäfer H, Bötzel K (2006). A randomized trial of deep-brain stimulation for Parkinson’s disease. N Engl J Med.

[B67] Williams A, Gill S, Varma T, Jenkinson C, Quinn N, Mitchell R (2010). Deep brain stimulation plus best medical therapy versus best medical therapy alone for advanced Parkinson’s disease (PD SURG trial): a randomised, open-label trial. Lancet Neurol.

[B68] Lee DJ, Lozano CS, Dallapiazza RF, Lozano AM (2019). Current and future directions of deep brain stimulation for neurological and psychiatric disorders. J Neurosurg.

[B69] Koeglsperger T, Palleis C, Hell F, Mehrkens JH, Bötzel K (2019). Deep brain stimulation programming for movement disorders: current concepts and evidence-based strategies. Front Neurol.

[B70] Castrioto A, Lozano AM, Poon Y-Y, Lang AE, Fallis M, Moro E (2011). Ten-year outcome of subthalamic stimulation in Parkinson disease: a blinded evaluation. Arch Neurol.

[B71] Ferreira JJ, Katzenschlager R, Bloem BR, Bonuccelli U, Burn D, Deuschl G (2013). Summary of the recommendations of the EFNS/MDS-ES review on therapeutic management of Parkinson’s disease. Eur J Neurol.

[B72] Munhoz RP, Picillo M, Fox SH, Bruno V, Panisset M, Honey CR (2016). Eligibility criteria for deep brain stimulation in Parkinson’s disease, tremor, and dystonia. Can J Neurol Sci.

[B73] Pollak P (2013). Deep brain stimulation for Parkinson’s disease -patient selection. Handb Clin Neurol.

[B74] Lhommée E, Wojtecki L, Czernecki V, Witt K, Maier F, Tonder L (2018). Behavioural outcomes of subthalamic stimulation and medical therapy versus medical therapy alone for Parkinson’s disease with early motor complications (EARLYSTIM trial): secondary analysis of an open-label randomised trial. Lancet Neurol.

[B75] Fox SH, Katzenschlager R, Lim S-Y, Barton B, de Bie RMA, Seppi K (2018). International Parkinson and movement disorder society evidence-based medicine review: update on treatments for the motor symptoms of Parkinson’s disease. Mov Disord.

[B76] DeLong MR, Huang KT, Gallis J, Lokhnygina Y, Parente B, Hickey P (2014). Effect of advancing age on outcomes of deep brain stimulation for Parkinson disease. JAMA Neurol.

[B77] Rossi M, Bruno V, Arena J, Cammarota Á, Merello M (2018). Challenges in PD patient management after DBS: a pragmatic review. Mov Disord Clin Pract.

[B78] Rughani A, Schwalb JM, Sidiropoulos C, Pilitsis J, Ramirez-Zamora A, Sweet JA (2018). Congress of neurological surgeons systematic review and evidence-based guideline on subthalamic nucleus and globus pallidus internus deep brain stimulation for the treatment of patients with Parkinson’s disease: executive summary. Neurosurgery.

[B79] Follett KA, Weaver FM, Stern M, Hur K, Harris CL, Luo P (2010). Pallidal versus subthalamic deep-brain stimulation for Parkinson’s disease. N Engl J Med.

[B80] Krack P, Volkmann J, Tinkhauser G, Deuschl G (2019). Deep brain stimulation in movement disorders: from experimental surgery to evidence-based therapy. Mov Disord.

[B81] Kalia LV, Lang AE (2015). Parkinson’s disease. Lancet.

[B82] Keus SHJ Munneke M (2007). Evidence-based analysis of physical therapy in Parkinson’s disease with recommendations for practice and research. Mov Disord.

[B83] Mak MKY, Hui-Chan CWY (2008). Cued task-specific training is better than exercise in improving sit-to-stand in patients with Parkinson’s disease: a randomized controlled trial. Mov Disord.

[B84] Monticone M, Ambrosini E, Laurini A, Rocca B, Foti C (2015). In-patient multidisciplinary rehabilitation for Parkinson’s disease: a randomized controlled trial. Mov Disord.

[B85] Nieuwboer A, Kwakkel G, Rochester L, Jones D, van Wegen E, Willems AM (2007). Cueing training in the home improves gait-related mobility in Parkinson’s disease: the RESCUE trial. J Neurol Neurosurg Psychiatry.

[B86] Lim I, van Wegen E, de Goede C, Deutekom M, Nieuwboer A, Willems A (2005). Effects of external rhythmical cueing on gait in patients with Parkinson’s disease: a systematic review. Clin Rehabil.

[B87] Ginis P, Nackaerts E, Nieuwboer A, Heremans E (2018). Cueing for people with Parkinson’s disease with freezing of gait: a narrative review of the state-of-the-art and novel perspectives. Ann Phys Rehabil Med.

[B88] Nutt JG, Bloem BR, Giladi N, Hallett M, Horak FB, Nieuwboer A (2011). Freezing of gait: moving forward on a mysterious clinical phenomenon. Lancet Neurol.

[B89] Rochester L, Baker K, Hetherington V, Jones D, Willems A-M, Kwakkel G (2010). Evidence for motor learning in Parkinson’s disease: acquisition, automaticity and retention of cued gait performance after training with external rhythmical cues. Brain Res.

[B90] Strouwen C, Molenaar EALM, Münks L, Keus SHJ, Zijlmans JCM, Vandenberghe W (2017). Training dual tasks together or apart in Parkinson’s disease: results from the DUALITY trial. Mov Disord.

[B91] Frazzitta G, Maestri R, Uccellini D, Bertotti G, Abelli P (2009). Rehabilitation treatment of gait in patients with Parkinson’s disease with freezing: a comparison between two physical therapy protocols using visual and auditory cues with or without treadmill training. Mov Disord.

[B92] Canning CG, Sherrington C, Lord SR, Close JCT, Heritier S, Heller GZ (2015). Exercise for falls prevention in Parkinson disease: a randomized controlled trial. Neurology.

[B93] Conradsson D, Löfgren N, Nero H, Hagströmer M, Ståhle A, Lökk J (2015). The effects of highly challenging balance training in elderly with Parkinson’s disease: a randomized controlled trial. Neurorehabil Neural Repair.

[B94] Capato TTC, de Vries NM, IntHout J, Barbosa ER, Nonnekes J, Bloem BR (2020). Multimodal balance training supported by rhythmical auditory stimuli in Parkinson’s disease: a randomized clinical trial. J Parkinsons Dis.

[B95] Capato TTC, de Vries NM, IntHout J, Ramjith J, Barbosa ER, Nonnekes J (2020). Multimodal balance training supported by rhythmic auditory stimuli in Parkinson disease: effects in freezers and nonfreezers. Phys Ther.

[B96] Capato TTC, Nonnekes J, de Vries NM, IntHout J, Barbosa ER, Bloem BR (2020). Effects of multimodal balance training supported by rhythmical auditory stimuli in people with advanced stages of Parkinson’s disease: a pilot randomized clinical trial. J Neurol Sci.

[B97] Ortelli P, Ferrazzoli D, Bera R, Caremani L, Giladi N, Maestri R (2018). Effectiveness of a goal-based intensive rehabilitation in Parkinsonian patients in advanced stages of disease. J Parkinsons Dis.

[B98] Schenkman M, Moore CG, Kohrt WM, Hall DA, Delitto A, Comella CL (2018). Effect of high-intensity treadmill exercise on motor symptoms in patients with de novo Parkinson disease: a phase 2 randomized clinical trial. JAMA Neurol.

[B99] Van der Kolk NM, de Vries NM, Kessels RPC, Joosten H, Zwinderman AH, Post B (2019). Effectiveness of home-based and remotely supervised aerobic exercise in Parkinson’s disease: a double-blind, randomised controlled trial. Lancet Neurol.

[B100] Manor Y, Mootanah R, Freud D, Giladi N, Cohen JT (2013). Video-assisted swallowing therapy for patients with Parkinson’s disease. Parkinsonism Relat Disord.

[B101] Atkinson-Clement C, Sadat J, Pinto S (2015). Behavioral treatments for speech in Parkinson’s disease: meta-analyses and review of the literature. Neurodegener Dis Manag.

[B102] Rao AK (2010). Enabling functional independence in Parkinson’s disease: update on occupational therapy intervention. Mov Disord.

[B103] Sturkenboom IHWM, Graff MJL, Hendriks JCM, Veenhuizen Y, Munneke M, Bloem BR (2014). Efficacy of occupational therapy for patients with Parkinson’s disease: a randomised controlled trial. Lancet Neurol.

[B104] Tofani M, Ranieri A, Fabbrini G, Berardi A, Pelosin E, Valente D (2020). Efficacy of occupational therapy interventions on quality of life in patients with parkinson’s disease: a systematic review and meta-analysis. Mov Disord Clin Pract.

